# 4407 Adapting CAPABLE as CAPABLE-FAMILY for the caregiver-care recipient dyad with Alzheimer’s dementia

**DOI:** 10.1017/cts.2020.367

**Published:** 2020-07-29

**Authors:** Emerald Rivers, Janiece Taylor, Erika Hornstein, Sarah Szanton

**Affiliations:** 1 Johns Hopkins University

## Abstract

OBJECTIVES/GOALS: The purpose of this 4-phase exploratory study is to create a human-centered protocol for a new program, CAPABLE-FAMILY, to address older adults with physical disability and dementia and their caregivers. METHODS/STUDY POPULATION: The Szanton-Gill Resilience Model, Verbrugge & Jett Disablement, and Life Span Theory of Control are theoretical frameworks guiding this study. **Phase 1.** Using qualitative research (n = 10 dyads) methods (ex. Photovoice) we seek to understand the context for older adults with dementia regarding disability. **Phase 2.** Using synthesis/ideation (n = 10 dyads) tools (ex. Journey Maps) we will synthesize the qualitative research during group ideation sessions with stakeholders. **Phase 3.** Using prototype testing (n = 3 dyads) methods (ex. Semantic Differentials, Storyboards), we will build the most promising prototypes. **Phase 4.** Using an open-label pilot (n = 3 dyads), we will test the interventions. RESULTS/ANTICIPATED RESULTS: We will assess disability (ADL, IADL), environmental changes pain, depression, polypharmacy, provider communication needs, and caregiver burden. We will interview the dyads and multiple CAPABLE Registered Nurses (RN), Occupational Therapists (OT), and Handymen (HM) about their prior experiences and perceptions of the pilot. CFQ, MocA, pain (BPI), and ZBI will be measured pre/post RN, OT, HM visit. While there are evidence-based programs to separately address cognitive impairment and physical disability, we anticipate this is the first study to develop a community-based goal-directed, human-centered approach for the maintenance of physical function for persons with dementia in the home. DISCUSSION/SIGNIFICANCE OF IMPACT: Persons with more cognitive impairment did not improve as much as those with less cognitive impairment in the original CAPABLE study, an evidenced based program which reduced disability. This suggests the need to adapt CAPABLE to reduce the burden of disability in persons with Alzheimer’s dementia. CONFLICT OF INTEREST DESCRIPTION: None

